# Pharmacological Manipulation of DNA Methylation in Adult Female Rats Normalizes Behavioral Consequences of Early-Life Maltreatment

**DOI:** 10.3389/fnbeh.2018.00126

**Published:** 2018-06-29

**Authors:** Samantha M. Keller, Tiffany S. Doherty, Tania L. Roth

**Affiliations:** Department of Psychological and Brain Sciences, University of Delaware, Newark, DE, United States

**Keywords:** epigenetics, DNMT, DNA methylation, development, maltreatment, early-life stress, females, behavioral outcomes

## Abstract

Exposure to adversity early in development alters brain and behavioral trajectories. Data continue to accumulate that epigenetic mechanisms are a mediating factor between early-life adversity and adult behavioral phenotypes. Previous work from our laboratory has shown that female Long-Evans rats exposed to maltreatment during infancy display both aberrant forced swim behavior and patterns of brain DNA methylation in adulthood. Therefore, we examined the possibility of rescuing the aberrant forced swim behavior in maltreated-adult females by administering an epigenome-modifying drug (zebularine) at a dose previously shown to normalize DNA methylation. We found that zebularine normalized behavior in the forced swim test in maltreated females such that they performed at the levels of controls (females that had been exposed to only nurturing care during infancy). These data help link DNA methylation to an adult phenotype in our maltreatment model, and more broadly provide additional evidence that non-targeted epigenetic manipulations can change behavior associated with early-life adversity.

## Introduction

The period after birth is a sensitive period during which environmental experiences are capable of altering the trajectory of brain development (Greenough et al., 1987; Rice and Barone, 2000; Knudsen, 2004). Exposure to adversity during this time has lifelong implications for the brain and, consequently, behavior (Fride et al., 1985; Heim et al., 1997; Brunson et al., 2001; McEwen, 2003; Lupien et al., 2009; Roth et al., 2009; Blaze and Roth, 2013, 2017; Blaze et al., 2013; Doherty et al., 2017). Caregiver maltreatment is a form of early-life adversity incurred by 10%–15% of the population in the United States (Gilbert et al., 2009; Lutz and Turecki, 2014). Individuals with a history of maltreatment are more likely to experience cognitive deficits such as problems with learning and memory (Rogosch et al., 1995; Pears and Fisher, 2005; De Bellis et al., 2013). Further, exposure to maltreatment confers susceptibility to developing psychiatric disorders including major depression, schizophrenia, and posttraumatic stress disorder (Beers and De Bellis, 2002; Cicchetti and Toth, 2005; Schenkel et al., 2005; Shea et al., 2005; Heim and Binder, 2012; Provencal and Binder, 2015).

Our laboratory implements a rodent model of caregiver maltreatment (Blaze and Roth, 2013, 2017; Blaze et al., 2013, 2015; Doherty et al., 2016, 2017) to better understand the consequences of early adversity on brain and behavioral development. Previous work from our lab has shown that female rodents exposed to brief bouts of daily caregiver maltreatment exhibit as adults mild deficits in novel object recognition (NOR) and an increased latency to become immobile in the forced swim test (Doherty et al., 2017). Interestingly, when animals were tested on these behavioral assays in adolescence, no differences were observed in behavioral performance between animals with a history of maltreatment relative to animals with a history of nurturing care in infancy (Doherty et al., 2017).

One way through which these early-life experiences might induce long-term consequences on behavior is via epigenetic alterations (Weaver et al., 2004; Champagne et al., 2006; Murgatroyd et al., 2009; Roth et al., 2009; Heim and Binder, 2012; Roth, 2012; Kundakovic et al., 2013; Lewis and Olive, 2014; McGowan and Roth, 2015; Silberman et al., 2016; Blaze et al., 2017). Epigenetic alterations, which are changes to chromatin that are capable of influencing gene expression without altering the underlying genomic sequence, include DNA methylation and posttranslational histone modifications (Attwood et al., 2002; Kouzarides, 2007; Li et al., 2007). Our lab has uncovered a number of epigenetic alterations throughout the adult brain of female subjects with a history of caregiver maltreatment, including increased DNA methylation of the brain-derived neurotrophic factor (*Bdnf*) gene in the hippocampus and prefrontal cortex coinciding with reduced methylation in the amygdala (Roth et al., 2009, 2014; Blaze and Roth, 2013, 2017; Blaze et al., 2013, 2015; Doherty et al., 2016). These same brain regions are recruited for behaviors known to be aberrant as a result of maltreatment (Duncan et al., 1986, 1993; Drevets et al., 1997; Blair, 2008; Zierhut et al., 2010; Antunes and Biala, 2012; Warburton and Brown, 2015), suggesting that these neurobiological changes could be involved in maltreatment-induced phenotypes. However, the extent to which epigenetic modifications resulting from exposure to maltreatment contribute to the altered behavioral trajectories in these animals is unknown. The goal of this study was to determine the ability of some phenotypic outcomes associated with caregiver maltreatment to be normalized by altering adult DNA methylation. Further, a positive finding would lend support to our hypothesis that the epigenetic changes resulting from early-life maltreatment are linked to phenotypic outcomes.

Manipulating the adult epigenome has the capacity to rescue outcomes of stress in rodents, with some supporting evidence for this in humans. For example, previous work from our lab demonstrated the capability of administration of zebularine (at the same dose used in this study), a drug known to modify DNA methylation, to normalize *Bdnf* DNA methylation and gene expression in the prefrontal cortex of animals with a history of maltreatment (Roth et al., 2009). Further, in clinical studies individuals benefitting from pharmaceutical or therapeutic interventions demonstrate epigenetic changes in peripheral tissues (Lopez et al., 2013; Perroud et al., 2013). The current study aimed to expand upon previous findings from our lab and explore the implications of manipulating DNA methylation on behavioral outcomes of maltreatment in adult subjects. Because the phenotypes under investigation were found in female, but not male, subjects exposed to caregiver maltreatment (Doherty et al., 2017), only female subjects were utilized in the current study.

## Materials and Methods

### Subjects and Infant Manipulations

All animal procedures were performed with approval from the University of Delaware Institutional Animal Care and Use committee following NIH established guidelines. Long-Evans rats (Blue Spruce outbred strain, obtained from Envigo) were bred in-house. Animals were given *ad libitum* access to food (Lab Diet 5P00, Prolab RMH 3000) and water, maintained on a 12-h light/dark cycle, and housed with Beta Chip heat-treated laboratory bedding. Multiparous dams (between the ages of 90 days and 1 year) were used for generating experimental litters and as stimulus dams. Fourteen total experimental litters were used in the study, derived from 10 different dams. The same breeder male (between the ages of 90 days and 1 year) and dam were only bred together one time so that each experimental litter had a different mother and father combination to promote genetic diversity in our experimental sample. Day of parturition was deemed postnatal day (PN) 0. On PN1, litters were culled to 12 pups (6 males and 6 females) when possible.

The scarcity-adversity model of low nesting resources was used as previously described (Roth et al., 2009; Doherty et al., 2016, 2017; Blaze and Roth, 2017; Walker et al., 2017). These manipulations were performed for 30 min daily from PN1–7. Briefly, we employed a within litter design whereby 1/3 of the experimental litter was exposed to maltreatment outside of the home cage (in a separate room from the vivarium), 1/3 of the same litter was cross-fostered to a nurturing dam outside of the home cage (in a separate room from the vivarium), and the remaining 1/3 of the litter remained in the home cage with the biological dam (in the vivarium). Pups from the cross-foster and maltreatment conditions were returned to the home cage with the biological dam immediately after the 30-min manipulations. The pups of the stimulus dams were placed into an incubator while the dams were being used for the experiment. Several (2–3 each) maltreatment and cross-foster dams were used for each weeklong experiment so that the same dam was not used for each day of the experiment. All dams (providing experimental litters or serving as maltreatment or cross-foster dams) were matched in postpartum age and diet, as it has been demonstrated that pups cannot distinguish between dams fed the same diet (Leon, 1975). The time of day the manipulations were conducted varied so that this would be an unpredictable stressor for dams and pups in the maltreatment condition. However, manipulations were always conducted during the light cycle from 7 AM to 7 PM.

For the maltreatment condition, the dam was given limited nesting resources (100 ml of Beta Chip bedding scattered on chamber floor) to care for the infant rats in an unfamiliar environment (45.5 × 30.5 × 45 cm opaque Plexiglas chamber). The cross-foster dam was given ample nesting resources (2–3 cm of Beta Chip bedding covering the chamber floor) and given 1 h to habituate to the environment (45.5 × 30.5 × 45 cm opaque Plexiglas chamber) prior to receiving infant rats. Videos of these infant manipulations were recorded. To confirm the efficacy of this model, behavioral videos of the infant manipulations from five (selected using a random number generator) of the 14 litters from which experimental subjects were collected were coded for nurturing (i.e., nursing, licking and grooming the pups) and adverse (i.e., actively avoiding, roughly handling, stepping on, dropping and dragging the pups) behaviors by trained scorers (89.5% inter-rater reliability). Each occurrence of a behavior was coded in 5-min time bins.

At the time of weaning, female rats were placed into cages of two or three animals from the same litter and infant condition. Only female offspring were used for behavioral testing in this study. Male subjects were utilized in each of the infant conditions and remained with the dam as part of the litter until weaning to eliminate the possibility of same-sex litter composition altering maternal behavior (Moore and Morelli, 1979; Hao et al., 2011; Kosten et al., 2014). Males were used for other experiments in our lab.

### Stereotaxic Surgery

Between PN70–80, stereotaxic surgery was performed. Anesthesia was induced using 5% isoflurane in oxygen. Following induction of anesthesia, animals were administered 2 mL of sterile saline and 0.03 mg/kg buprenorphine. Animals were subsequently placed into a stereotaxic frame and anesthesia was maintained using 2%–3% isoflurane in oxygen. A stainless steel guide cannula (22 gauge, 8 mm length, Plastics One Inc., Roanoke, VA, USA) was implanted into the left lateral ventricle using the following coordinates: 1.5 mm posterior, 2.0 mm lateral, and 3.0 mm ventral relative to bregma. At the time of surgery, cannula placement was verified using gravitational saline let-down as has been done in other reports (Asok et al., 2016). Once surgery was completed, a dummy cannula extending 1 mm beyond the guide cannula was inserted into the guide cannula. Animals were single-housed following surgery to avoid injury. In a subset of animals, cannula tracks were also visually confirmed postmortem after slicing brains in a cryostat at −12°C.

### Drug Administration

After 1 day of recovery from stereotaxic surgery, intracerebroventricular (ICV) infusion of either zebularine or vehicle began as has been previously described (Roth et al., 2009). Zebularine, which is a cytidine analog, incorporates into DNA and prevents DNA methyltransferases (DNMTs) from adding methyl groups to DNA (Champion et al., 2010; Gnyszka et al., 2013). This drug has previously been demonstrated to alter levels of DNA methylation in the brains of adult animals (Lubin et al., 2008; Roth et al., 2009, 2015; Anier et al., 2010). Zebularine (Sigma Aldrich) was dissolved in dimethyl sulfoxide (DMSO) and subsequently diluted with sterile saline such that the solution was comprised of 10% DMSO. Zebularine was administered via an infusion cannula (28 gauge) attached to PE20 tubing at a dose of 600 ng/μl delivered at the rate of 1 μl per minute (2 μl volume). This dose was selected because it has been previously demonstrated to reverse maltreatment-induced DNA methylation of the *Bdnf* gene (Roth et al., 2009). An equivalent amount of vehicle (10% DMSO in sterile saline) was administered at the same rate. Previous studies have shown 10% DMSO to be an acceptable concentration and not cause cell toxicity or aberrations within neurons (Da Violante et al., 2002; Soltani et al., 2016). Zebularine or vehicle solution was administered once daily for 7 days.

### Adult Behavior

**Figure 1:**
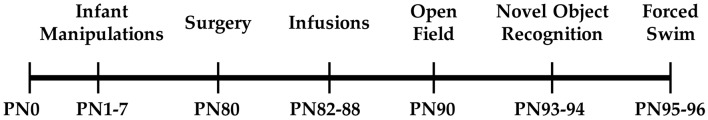
Timeline of experimental procedures implemented in this study.

One day after the final drug or vehicle infusion, behavioral testing commenced. Animals were run through open-field, NOR, and forced swim testing following protocols previously implemented by our lab and others (Arakawa, 2003; Oliveira et al., 2010; Castagné et al., 2011; Doherty et al., 2017) and briefly described below. A timeline of experimental procedures can be seen in Figure 1. Testing was conducted in a room with white noise under red light. A camera placed on the ceiling above the behavioral apparatuses and Any-maze software (Stoelting Co., Wood Dale, IL, USA) were used to record all behavioral procedures. After behavioral testing, a subset of rats were sacrificed and their brains were harvested for slicing. Post-mortem vaginal lavages were performed to determine estrous cycle stage for all females.

### Open-Field Testing

Rats were placed into a circular arena (84 cm diameter × 36 cm height) for 10 min. This test (as well as for all other tests) was conducted in a dark room under red light, as has been previously conducted by our lab (Doherty et al., 2017). Any-maze software (Stoelting Inc.) was used to score behavior in open-field testing. Time spent in the center of the field, number of entries into the center, and distance traveled were all recorded. The apparatus was cleaned using 70% ethanol in between subjects.

### Novel Object Recognition

Novel object testing occurred in the same apparatus utilized for open-field testing. Animals were habituated to this chamber for an additional 2 days following open-field testing. Subjects were exposed to two identical objects for 15 min. Binder clips and conical tubes were used as objects (Doherty et al., 2017). Total time spent exploring the items was recorded. Twenty-four hours later, rats were exposed to one item from the previous day in addition to a novel item. Objects used and their placement in the apparatus were counterbalanced across experimental subjects. To quantify the amount of time the rats spent exploring the novel object, a ratio was computed whereby the total time spent exploring the novel object was divided by the total time spent exploring both the novel and familiar objects. Behavior was recorded using Any-maze software and later scored offline by trained scorers blind to experimental groups.

### Forced Swim

One day after NOR, rats were placed into a bucket (29 cm diameter × 48 cm height) of 25°C water and given 15 min to swim. Once rats were removed from the water, they were dried using a microfiber cloth and their cage was placed on a heating pad under a heat lamp until they were completely dry. Twenty-four hours later, animals were returned to the water bucket for a 5-min test. Behavior was recorded using Any-maze software and later scored offline by trained scorers blind to experimental conditions. Time spent immobile, which was defined as performing only the motions necessary to keep the head above water, and latency until the first bout of immobility were coded. Two animals were removed from the data set for neglecting to demonstrate immobility during the forced swim test (one normal maternal care vehicle subject and one cross-foster care zebularine subject).

### Statistical Analyses

GraphPad Prism 7.03 software was used for all statistical analyses. Caregiving behavior data were analyzed using a two-way ANOVA (levels: caregiving behavior (i.e., nurturing and adverse), infant condition). Two-way ANOVAs (levels: infant condition, drug or vehicle) were used for analyzing behavioral data. For all analyses, *p* < 0.05 was used to denote statistical significance. A chi-squared analysis was conducted on estrus cycle stage of subjects. *T*-tests were used for *post hoc* analyses and Bonferroni corrections were applied where necessary.

## Results

### Infant Manipulations

**Figure 2:**
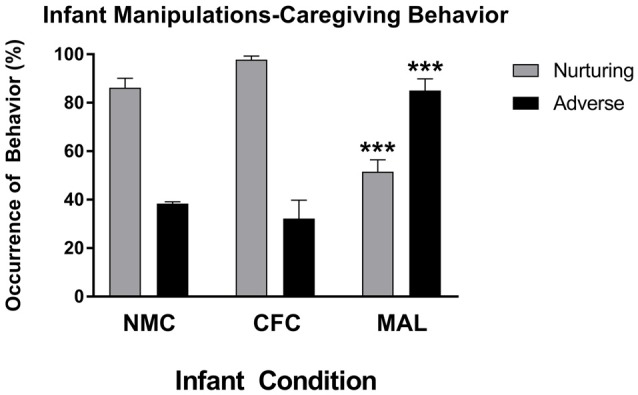
Infant rats exposed to the maltreatment condition received more adverse and less nurturing behavior relative to infant rats assigned to the cross-foster and normal maternal care conditions. NMC, normal maternal care; CFC, cross-foster care; MAL, maltreatment. *n* = 5 dams; error bars represent SEM; *** denotes *p* < 0.0001.

Two-way ANOVAs performed on nurturing and aversive care observed across our infant conditions revealed a main effect of caregiving behavior (*F*_(2,24)_ = 67.74, *p* < 0.0001) and a significant interaction of caregiving behavior and infant manipulation condition (*F*_(1,24)_ = 51.72, *p* < 0.0001), which is consistent with findings from other reports using this form of the scarcity-adversity model (Roth et al., 2009, 2014; Blaze and Roth, 2013; Hill et al., 2014; Doherty et al., 2016, 2017). As illustrated in Figure 2, *post hoc* analyses revealed that significantly more adverse behaviors were observed in the maltreatment condition relative to the cross-foster (*p* < 0.0001) and normal maternal (*p* < 0.0001) care conditions, while more nurturing behaviors were performed in the cross-foster (*p* < 0.0001) and normal maternal care (*p* = 0.0002) conditions relative to the maltreatment condition. No differences in nurturing (*p* = 0.4797) or adverse care (*p* = 0.9242) were observed between the cross-foster and normal care conditions. More nurturing care was observed relative to adverse care in both normal care (*p* < 0.0001) and cross-foster care (*p* < 0.0001) conditions, while more adverse care was observed relative to nurturing care in the maltreatment condition (*p* = 0.0003).

### Estrus Cycle

A chi-squared analysis revealed that there were no significant differences in estrus cycle (i.e., estrus, diestrus day 1, diestrus day 2, and proestrus) status across experimental groups (*χ*^2^_(15, *N* = 71)_ = 20.8, *p* = 0.1688).

### Open-Field Testing

**Figure 3:**
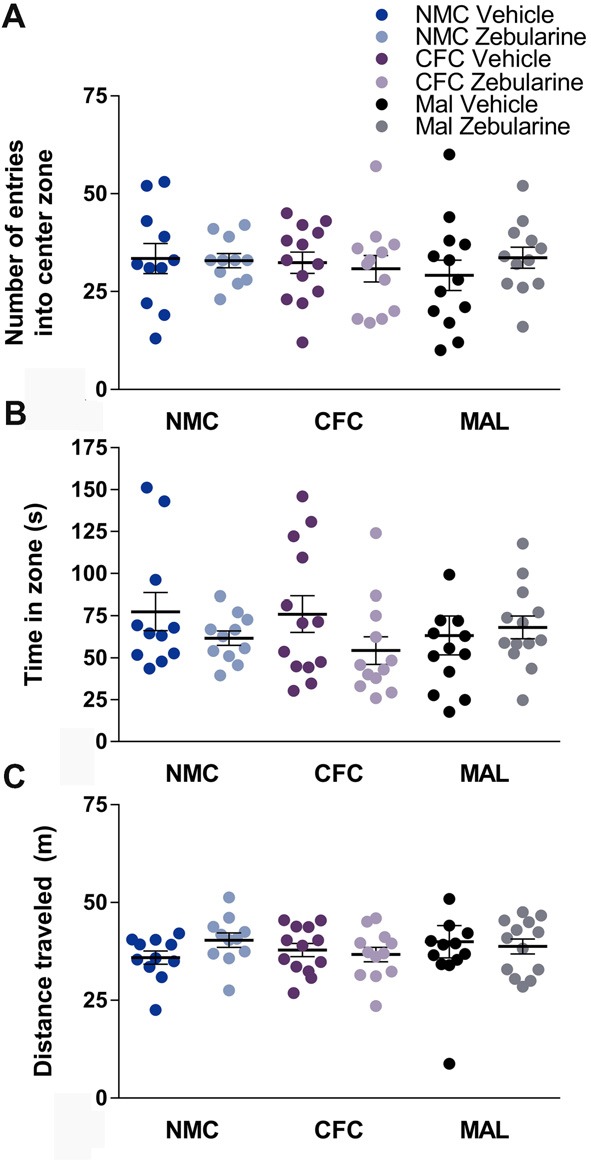
No differences were found as a result of infant condition or drug treatment on number of entries into the center zone **(A)** time spent in zone **(B)** or distance traveled **(C)** in the open-field test. NMC, normal maternal care; CFC, cross-foster care; MAL, maltreatment. *n* = 11–13/group; error bars represent SEM.

No statistically significant effects of infant caregiver condition or drug treatment were observed in the open-field test on any of the measures recorded including entries into the center (Figure 3A), time spent in the center (Figure 3B), or distance traveled (Figure 3C) within the behavioral apparatus (*p*’s > 0.05). Behavior did change over time in the open field test, however, the analyses did not reveal any group differences (*p*’s > 0.05). Specifically, animals regardless of drug treatment or infant condition traveled less distance over time during the test (*p* < 0.0001) and made less center entries across time (*p* = 0.0009). Time in the center did not change over time (*p* = 0.2929). These results suggest that locomotor behavior was not altered as a result of infant caregiver or drug treatment condition.

### Novel Object Recognition

**Figure 4:**
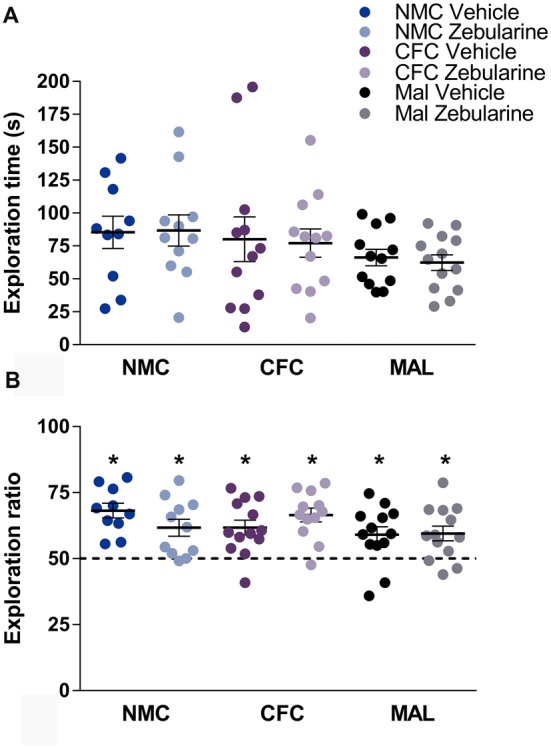
We did not find an effect of infant caregiver history or zebularine treatment on time spent investigating objects **(A)** or novel-object recognition testing **(B)**. Subjects from all conditions were able to perform novel object recognition (NOR). Dashed line at 50% indicates chance performance. NMC, normal maternal care; CFC, cross-foster care; MAL, maltreatment. *n* = 11–13/group; error bars represent SEM; * denotes *p* < 0.05.

Neither infant caregiver condition nor drug treatment had a statistically significant effect on total time spent exploring the objects during habituation (Figure 4A, *p*’s > 0.05). There was likewise no significant effect of drug treatment or infant caregiver condition on the novel-to-familiar object ratio (Figure 4B, *p*’s > 0.05). As demonstrated by *t*-tests performed relative to chance (i.e., 50% exploration time with the novel object), subjects from all conditions were able to perform NOR (*p*’s < 0.05).

### Forced Swim

**Figure 5:**
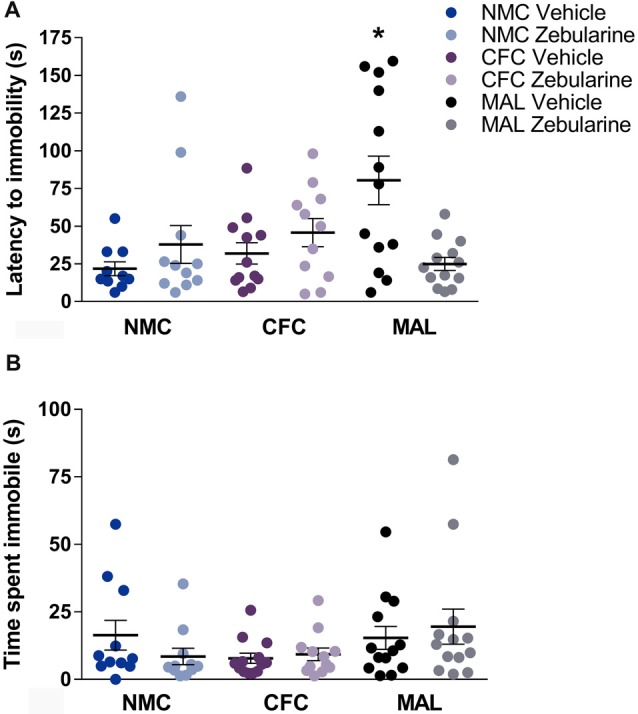
Female rats with a history of maltreatment demonstrated an increased latency to become immobile in the forced swim test **(A)** Treatment with zebularine normalized this behavior, but did not alter behavior in females with a history of nurturing maternal care. No differences were found as a result of infant manipulation or drug treatment on total time spent immobile **(B)** NMC, normal maternal care; CFC, cross-foster care; MAL, maltreatment. *n* = 11–13/group; error bars represent SEM; * denotes *p* = 0.0007.

For latency to immobility, a significant interaction was found between drug treatment and infant condition (*F*_(2,64)_ = 8.234, *p* = 0.0007). *Post hoc* analyses revealed that the maltreatment group administered vehicle was significantly different from the normal (*p* = 0.0027) and cross-foster care (*p* = 0.0146) vehicle-treated control groups (Figure 5A). Further, the zebularine-treated animals with a history of maltreatment were significantly different from their vehicle-treated counterparts (*p* = 0.0022). This suggests that animals with maltreatment history display altered behavior (latency to immobility) in the forced swim test and zebularine administration in adulthood was able to normalize this behavior measure. No statistically significant differences were found as a result of drug treatment or infant condition on total time spent immobile (Figure 5B, *p*’s > 0.05).

## Discussion

We replicated our previous finding that exposure to adverse caregiving conditions in infancy induces alterations in forced swimming behavior in adult females (Doherty et al., 2017). Specifically, animals with a history of maltreatment took longer to demonstrate their first bout of immobility relative to animals with a history of nurturing care. The main finding from the current study was that administration of zebularine to maltreated-animals at a dose previously shown to decrease DNA methylation and rescue gene expression (Roth et al., 2009) was capable of normalizing this behavior, with no effect on forced swim behavior in animals with a history of nurturing care.

Several interpretations of behavior in the forced swim test have been proposed (Porsolt et al., 1977, 1978; Nishimura et al., 1988; De Pablo et al., 1989; West, 1990; de Kloet and Molendijk, 2016). One interpretation is that animals that learn to go immobile during this inescapable swim stressor are exhibiting an adaptive coping strategy, as immobility would allow for energy conservation (West, 1990; Andolina et al., 2015; de Kloet and Molendijk, 2016; Campus et al., 2017). Using this interpretation of the test, our data would suggest that animals with a history of maltreatment are failing to exhibit adaptive coping when faced with a stressful situation. This interpretation would be consistent with other reports finding impairments in coping in individuals with a history of early-life stress (Dich et al., 2015; Wadsworth, 2015; Grace et al., 2017). Deficits in the forced-swim task may also be reflective of an inability to learn the immobility behavior (De Pablo et al., 1989). Using this interpretation, our data would suggest that animals with a history of maltreatment exhibit an inability to readily learn this behavior. This interpretation would be consistent with other reports of learning impairments in animals exposed to early adversity (Doherty et al., 2017; Walker et al., 2017). A reduction in the latency to immobility in the maltreated-animals that received zebularine could suggest that altering aberrant methylation levels promoted an adaptive coping strategy and/or facilitated learning of immobility behavior. Finally, it should be noted that the forced swim test is often used to screen for depressive-like behavior and the effects of antidepressant drugs on these behaviors, with antidepressants for example typically increasing the latency to first immobility (Porsolt et al., 1977, 1978). Using this interpretation, zebularine administration might be producing an undesirable effect in animals with a history of maltreatment. Though this study was not designed to parse out these interpretations, our data nonetheless demonstrate the ability of infusions of zebularine to change a phenotype associated with exposure to early adversity. Measures such as escape and mild swimming would be important variables to include in a future study to help understand the functional significance of this phenotype.

Previous work from our lab found a mild but significant deficit in NOR in adult female animals with a history of maltreatment (Doherty et al., 2017). While our maltreated animals did not exhibit a statistically significant deficit in NOR, this group did show a numerically lower novel object preference and more variability in their performance (*M* = 59.01, *SD* = 10.99) relative to vehicle-treated normal care animals (*M* = 68.16, *SD* = 8.77). While we neglected to replicate the modest deficit in NOR in females with a history of maltreatment, several methodological differences exist between our previous study and the current study that could explain this discrepancy. Due to our drug treatment regimen, animals here received daily handling for 1 week, whereas in our previous work animals were handled at most three times prior to the behavioral assays. Further, in our previous study, animals were pair-housed, while in the current study animals were single-housed immediately following surgery (to aid in recovery from surgery).

Interestingly, a reduction in novel object preference and increased variability was observed in our drug-treated normal care animals (*M* = 61.69, *SD* = 10.63), as about half the subjects in this group did not exhibit a novel object preference while all of the vehicle-treated normal maternal care animals did exhibit a preference. The lack of NOR performance in some of the zebularine-treated normal maternal care animals is consistent with a previous report finding an impairment in NOR in normal animals after administration of a DNMT inhibitor (Scott et al., 2017). Future research is warranted to elucidate the reasons for the variability in NOR performance and the effect, if any, of zebularine treatment and exposure to maltreatment on the ability to perform NOR in individual subjects.

In conclusion, data presented here are consistent with the hypotheses that epigenetic alterations produced by exposure to adverse caregiving conditions play a role in adult phenotypes and that non-targeted epigenetic manipulations can change behavior. The current study is one of few that have investigated the possibility of altering the female epigenome in adulthood (for review see; Keller and Roth, 2016). While reports have found an effect of zebularine administration on behavior in adult rats (Lubin et al., 2008; Roth et al., 2015), to the knowledge of the authors there are no data on the duration of time that the effects of zebularine administration are maintained. The results of the current study suggest that zebularine is able to impact behavior for at least 1 week after administration, as the forced swim test was conducted 7 days after the final zebularine infusion. While our results suggest that altering brain DNA methylation has implications for behavior, it is unclear which genes and which brain regions are contributing to the observed behavioral effects and whether zebularine may be having any non-specific drug effects, all of which warrant elucidation in future research.

## Author Contributions

SK and TR designed the study. SK and TD performed experiments and data analyses. All authors took part in interpretation of the results. SK wrote the first draft of the manuscript. TD and TR edited the manuscript.

## Conflict of Interest Statement

The authors declare that the research was conducted in the absence of any commercial or financial relationships that could be construed as a potential conflict of interest.
